# Stability and consistency of symptom clusters in younger versus older patients receiving chemotherapy

**DOI:** 10.1186/s12877-024-04755-2

**Published:** 2024-02-16

**Authors:** Lisa Morse, Bruce A. Cooper, Christine S. Ritchie, Melisa L. Wong, Kord M. Kober, Carolyn Harris, Joosun Shin, Kate Oppegaard, Marilyn J. Hammer, Alejandra Calvo Schimmel, Steven M. Paul, Yvette P. Conley, Jon D. Levine, Christine Miaskowski

**Affiliations:** 1 School of Nursing, University of California, San Francisco, CA USA; 2 Division of Palliative Care and Geriatric Medicine, Massachusetts General Hospital Morgan Institute, Boston, MA USA; 3 School of Medicine, University of California, San Francisco, CA USA; 4 Division of Research, Kaiser Permanente Medical Group, Oakland, CA USA; 5 School of Nursing, University of Pittsburgh, Pittsburgh, PA USA; 6 Dana Farber Cancer Institute, Boston, MA USA; 7 Department of Physiological Nursing, School of Nursing, University of California, 2 Koret Way– N631Y, 94143-0610 San Francisco, CA USA

**Keywords:** Cancer, Chemotherapy, Exploratory factor analysis, Older adults, Patient reported outcomes, Symptoms, Symptom clusters

## Abstract

**Background:**

By 2035, the number of newly diagnosed cancer cases will double and over 50% will be in older adults. Given this rapidly growing demographic, a need exists to understand how age influences oncology patients’ symptom burden. The study purposes were to evaluate for differences in the occurrence, severity, and distress of 38 symptoms in younger (< 60 years) versus older (≥ 60 years) oncology patients undergoing chemotherapy and to evaluate for differences in the stability and consistency of symptom clusters across the two age groups.

**Methods:**

A total of 1329 patients were dichotomized into the younger and older groups. Patients completed demographic and clinical questionnaires prior to the initiation of their second or third cycle of chemotherapy. A modified version of Memorial Symptom Assessment Scale was used to evaluate the occurrence, severity, and distress of 38 common symptoms associated with cancer and its treatment. Differences between the two age groups in demographic and clinical characteristics and ratings of occurrence, severity, and distress for the 38 symptoms were evaluated using parametric and nonparametric tests. Exploratory factor analyses were done within each age group to identify symptom clusters using symptom occurrence rates.

**Results:**

Compared to the younger group (14.8 (± 7.0)), older adults reported a lower mean number of symptoms (12.9 (± 7.2)). Older patients experienced lower occurrence rates for almost 50% of the symptoms. Regarding symptom clusters, an eight-factor solution was selected for both age groups. Across the two age groups, the eight symptom clusters (i.e., physical and cognitive fatigue, respiratory, psychological, hormonal, chemotherapy-related toxicity, weight gain, gastrointestinal, epithelial) were stable. However, symptoms within the physical and cognitive, chemotherapy-related toxicity, and gastrointestinal clusters were not consistent across the age groups.

**Conclusions:**

To be able to provide tailored and effective symptom management interventions to older oncology patients, routine assessments of the core symptoms unique to the symptom clusters identified for this group warrants consideration. The underlying mechanism(s) for these inconsistencies in symptom burden is an important focus for future studies.

## Background

By 2035, the number of newly diagnosed cancer cases will double and over 50% of these cases will be in older adults [1]. Given this rapidly growing demographic, a need exists to understand how age influences oncology patients’ symptom burden. Limited evidence suggests that a higher symptom burden in older oncology patients has a negative impact on treatment adherence [2], ability to perform activities of daily living [3], and overall quality of life [2, 4–6].

Only two studies have described age differences in the symptom experience of oncology patients before or during active treatment [7, 8]. In one study that used the Memorial Symptom Assessment Scale (MSAS) [7], compared to younger adults (< 60 years, *n* = 263), older adults (≥ 60 years, *n* = 330) reported lower occurrence rates for 15 out of 32 symptoms and lower severity and distress ratings for six and fourteen symptoms, respectively. Eight of the eleven symptoms with the highest occurrence rates were identical in the two age groups.

In another study that evaluated for differences in occurrence and severity ratings of fatigue, decrements in energy, and sleep disturbance in younger (< 65 years) versus older (≥ 65 years) oncology patients using the Lee Fatigue Scale and the General Sleep Disturbance Scale (GSDS) [8], fewer older adults had GSDS scores above the clinically meaningful cutoff score. In addition, older adults reported lower levels of morning and evening fatigue and higher levels of evening energy. While these studies provide important insights into differences in individual symptoms, given that older adults experience an average of nine to ten unrelieved symptoms [9], an equally important area for research is an examination of symptom clusters in older oncology patients.

Only two studies used exploratory factor analysis (EFA) to identify symptom clusters in older oncology patients [5, 6]. In a study of 220 patients with lung cancer, who ranged in age from 65 to 89 years [6], one symptom cluster with seven symptoms (i.e., nausea, fatigue, weakness, appetite loss, weight loss, altered taste, vomiting) was identified using the Physical Symptom Experience tool. In another study of 192 older (≥ 65 years) breast cancer survivors [5], using the Symptom Bother Scale-Revised, seven symptom clusters were identified (i.e., musculoskeletal, neurocognitive, dryness, urinary, circulatory, sleep, hormonal). These inconsistent findings may be due to differences in: sample characteristics, symptom measures, timing of the assessments, and/or the symptom dimensions evaluated.

Two studies reported on age differences in symptom clusters [10, 11]. In the first study [10], separate EFAs were done for younger (< 60 years, *n* = 263) and older (≥ 60 years, *n* = 330) patients using the occurrence ratings for the 32 MSAS symptoms. The authors used the term “partially concordant” to describe clusters with similar items (i.e., symptoms) and structure coefficients. Four symptom clusters were labeled partially concordant between the two groups (i.e., mood/cognitive, malaise, body image, genitourinary). Three symptom clusters were unique to the younger patients (i.e., treatment-related, gastrointestinal, hormonal). Three symptom clusters were unique to the older patients (i.e., aerodigestive, nutrition, age-related). The authors concluded that the clusters unique to the older group reflected age-related physiological changes. Symptom clusters unique to the younger group were considered treatment-related. Symptom clusters identified in the older patients typically included a larger and more diverse range of symptoms.

In another study that evaluated symptom clusters using the Edmonton Symptom Assessment System (ESAS) in younger (≤ 60 years, *n* = 538) versus older (> 60 years, *n* = 820) patients with advanced cancer [11], two common symptom clusters were identified. For both age groups, the primary symptom cluster consisted of pain, nausea, and decreased appetite. The second symptom cluster consisted of depression and anxiety. The small number of clusters identified may be related to the fact that the ESAS evaluates the severity of only nine physical and psychological symptoms. Neither of these studies reported on the stability and consistency of the symptom clusters across the two age groups [10, 11].

Given the paucity of research on age-related differences in the symptom experience of oncology patients and the inconsistent findings across the two comparative symptom cluster studies [10, 11], additional research is warranted. Therefore, the purposes of this study were to evaluate for differences in the occurrence, severity, and distress of 38 symptoms in younger (< 60 years) versus older (≥ 60 years) patients undergoing chemotherapy and evaluate for differences in the stability and consistency of symptom clusters across the two age groups. We hypothesized that older patients would have lower symptom occurrence, severity, and distress ratings and that common and distinct symptom clusters would be identified between the two age groups.

## Methods

### Patients and settings

This cross-sectional study is an analysis from a parent, descriptive, longitudinal study that evaluated the symptom experience of oncology outpatients receiving chemotherapy [12–14]. Eligible patients were ≥ 18 years of age; had a diagnosis of breast, lung, gastrointestinal, or gynecological cancer; had received chemotherapy within the preceding four weeks; were scheduled to receive at least two additional cycles of chemotherapy; were able to read, write and understand English; and gave written informed consent. Patients were recruited from two Comprehensive Cancer Centers, one Veteran’s Affairs hospital, and four community-based oncology programs from March 2010 to May of 2015.

### Study procedures

The study was approved by the Committee on Human Research at the University of California, San Francisco (#10-02885) and by the Institutional Review Board at each study site. Written informed consent was obtained from all patients. Patients completed questionnaires in their homes a total of six times over two cycles of chemotherapy. Data from the enrollment assessment (i.e., before the receipt of the second or third cycle of chemotherapy) were used in this analysis. Medical records were reviewed for disease and treatment information.

### Instruments

#### Demographic and clinical characteristics

Patients completed a demographic questionnaire, Karnofsky Performance Status (KPS) scale [15], Alcohol Use Disorders Identification Test (AUDIT) [16], and Self-Administered Comorbidity Questionnaire (SCQ). The SCQ evaluates the occurrence, treatments for, and impact of 13 common medical conditions [17]. The MAX-2 score was used to evaluate the toxicity of various chemotherapy regimens [18].

#### Symptom measure

A modified version of the 32-item MSAS was used to evaluate the occurrence, severity, and distress of 38 common symptoms associated with cancer and its treatment [19]. Six common symptoms were added: hot flashes, chest tightness, difficulty breathing, abdominal cramps, increased appetite, and weight gain. Using the MSAS [19], patients reported whether they had experienced each symptom in the past week. If they had experienced the symptom, they were asked to rate its severity and distress. Severity and distress were rated using four-point and five-point Likert scales, respectively.

### Data analyses

Patients were dichotomized to the younger (< 60 years) and older (≥ 60 years) groups based on the World Health Organization’s reference to the older population as being ≥ 60 years [20]. Differences between the two age groups in demographic and clinical characteristics, as well as for ratings of occurrence, severity, and distress for the 38 MSAS symptoms, were evaluated using parametric and nonparametric tests. A *p*-value of < 0.05 was considered statistically significant. These analyses were done using IBM SPSS Statistics version 29 (IBM Corporation, Armonk, NY).

To identify the symptom clusters, EFA was done using MPlus Version 8.8 [21]. Separate EFAs were done for each age group using patients’ ratings of the occurrence of the 38 MSAS symptoms. Factor loadings were considered meaningful if the loading was ≥ 0.40 [21]. Factors were adequately defined if at least two symptoms had loadings of ≥ 0.40 [22]. Items were allowed to cross-load if they fell within our preset criteria of ≥ 0.40. Tetrachoric correlations were used to create the matrix of associations for the occurrence items [21]. Simple structures for the EFAs were estimated using the method of unweighted least squares with geomin (i.e., oblique) rotation [21].

Factor solutions were estimated for two through eight factors. Factor solutions with the greatest interpretability and clinical meaningfulness were selected, given that they met the criteria set for evaluating simple structure. Clusters were named based on the symptoms with the highest factor loadings and the majority of the symptoms in the cluster.

### Evaluation of stability and consistency

As was done in our previous studies [12, 23], in the current study, the term *stability* describes whether or not the same clusters are identified across the two age groups. In contrast, *consistency* describes whether the specific symptoms within a cluster remain the same across the two age groups. For a cluster to be considered consistent, the two or three symptoms with the highest factor loadings must be present across both age groups. Given that a symptom cluster must contain a minimum of two symptoms [9], a minimum of the same two symptoms with the highest factor loadings should be applied to clusters with only two or three symptoms. For clusters with four or more symptoms, a minimum of the same three symptoms with the highest factor loadings must be present across the age groups to be considered consistent.

## Results

### Differences in demographic and clinical characteristics

A total of 2234 patients were approached and 1343 consented to participate (60.1% response rate) in the parent study. The primary reason for refusal was being overwhelmed with their cancer treatment. Of the 1343 patients enrolled, 1329 patients completed the occurrence ratings on the MSAS [19] that were used to create the symptom clusters.

In the current study, 55.2% of the patients were < 60 years and 44.8% were ≥ 60 years. As shown in Table 1, numerous demographic and clinical characteristics differed between the two age groups. In brief, compared to the younger group, the older group was less likely to be female, more likely to live alone, less likely to be employed, and had a lower annual income. In terms of clinical characteristics, the older group had a higher comorbidity burden, a poorer functional status score, a higher number of previous cancer treatments, a lower MAX2 score, and were more likely to have metastatic disease.

**Table 1:** Differences in Demographic and Clinical Characteristics Between the Two Age Groups at Enrollment

Characteristics	Younger (0) (< 60 years) 55.2% (*n* = 741)	Older (1) (≥ 60 years) 44.8% (*n* = 602)	Statistics
	Mean (SD)	Mean (SD)	
Age (years)	48.4 (8.5)	68.0 (6.2)	t=-49.06, *p* < 0.001
Education (years)	16.1 (3.0)	16.3 (3.1)	t=-0.88, *p* = 0.378
Body mass index (kg/m^2^)	26.1 (5.7)	26.4 (5.6)	t=-1.14, *p* = 0.256
Karnofsky Performance Status score	79.0 (12.3)	81.2 (12.6)	t=-3.15, *p* = 0.002
Number of comorbidities out of 13	2.1 (1.3)	2.8 (1.5)	t=-8.10, *p* < 0.001
Self-Administered Comorbidity Questionnaire score	5.0 (2.9)	6.1 (3.4)	t=-6.31, *p* < 0.001
Alcohol Use Disorders Identification Test score	3.0 (2.6)	3.0 (2.4)	t = 0.09, *p* = 0.929
Time since cancer diagnosis (years)	1.6 (3.1)	2.5 (4.6)	U, *p* < 0.001
Time since diagnosis (median)	0.40	0.46	
Number of prior cancer treatments	1.5 (1.5)	1.7 (1.5)	t=-2.41, *p* = 0.016
Number of metastatic sites excluding lymph node involvement	0.7 (1.1)	0.9 (1.0)	t=-2.53 *p* = 0.012
Number of metastatic sites, including lymph node involvement	1.2 (1.3)	1.3 (1.2)	t=-1.86, *p* = 0.063
MAX2 score	0.18 (0.08)	0.16 (0.08)	t = 5.31, *p* < 0.001
Number of MSAS symptoms out of 38	14.8 (7.2)	12.9 (7.0)	t = 4.85, *p* < 0.001
	% (n)	% (n)	
Female	83.9 (622)	70.2 (422)	FE, *p* < 0.001
Race/ethnicity			χ^2^ = 51.59, *p* < 0.001
White Asian or Pacific Islander Black Hispanic, Mixed, or Other	63.2 (461) 17.3 (126) 6.0 (44) 13.6 (99)	77.2 (460) 7.0 (42) 8.6 (51) 7.2 (43)	0 < 1 0 > 1 NS 0 > 1
Married or partnered (% yes)	65.7 (479)	63.0 (375)	FE, *p* = 0.327
Lives alone (% yes)	17.8 (130)	25.9 (154)	FE, *p* < 0.001
Childcare responsibilities (% yes)	36.1 (262)	4.8 (28)	FE, *p* < 0.001
Care of adult responsibilities (% yes)	9.1 (62)	6.5 (35)	FE, *p* = 0.110
Currently employed (% yes)	42.0 (308)	26.5 (158)	FE, *p* < 0.001
Annual household income			
<$30,000 $30,000 to <$70,000 $70,000 to <$100,000 >$100,000	17.1 (115) 18.9 (127) 16.2 (109) 47.7 (320)	20.0 (106) 24.0 (127) 17.7 (94) 38.3 (203)	U, *p* = 0.002
Specific comorbidities (% yes)			
Heart disease	2.0 (15)	10.3 (62)	FE, *p* < 0.001
High blood pressure	19.7 (146)	43.2 (260)	FE, *p* < 0.001
Lung disease	6.5 (48)	17.4 (105)	FE, *p* < 0.001
Diabetes	5.3 (39)	13.8 (83)	FE, *p* < 0.001
Ulcer or stomach disease	4.9 (36)	4.8 (29)	FE, *p* = 1.000
Kidney disease	0.9 (7)	2.0 (12)	FE, *p* = 0.162
Liver disease	5.8 (43)	7.3 (44)	FE, *p* = 0.268
Anemia or blood disease	14.7 (109)	9.1 (55)	FE, *p* = 0.002
Depression	19.4 (144)	18.8 (113)	FE, *p* = 0.781
Osteoarthritis	6.2 (46)	19.6 (118)	FE, *p* < 0.001
Back pain	25.5 (189)	26.1 (157)	FE, *p* = 0.851
Rheumatoid arthritis	2.3 (17)	4.3 (26)	FE, *p* = 0.042
Exercise on a regular basis (% yes)	74.6 (540)	66.0 (389)	FE, *p* < 0.001
Current or history of smoking (% yes)	26.3 (192)	46.6 (275)	FE, *p* < 0.001
Type of cancer			χ^2^ = 93.26, *p* < 0.001
Breast Gastrointestinal Gynecologic Lung	50.5 (374) 28.2 (209) 15.0 (111) 6.3 (47)	27.6 (166) 33.7 (203) 20.3 (122) 18.4 (111)	0 > 1 0 < 1 0 < 1 0 < 1
Type of prior cancer treatment			χ^2^ = 25.73, *p* < 0.001
No prior treatment Only surgery, CTX, or RT Surgery & CTX, or Surgery & RT, or CTX & RT Surgery & CTX & RT	27.0 (196) 45.1 (327) 15.0 (109) 12.8 (93)	22.2 (129) 38.3 (222) 25.9 (150) 13.6 (79)	0 > 1 0 > 1 0 < 1 NS
Metastatic sites			χ^2^ = 24.02, *p* < 0.001
No metastasis Only lymph nodes Only non-lymph nodes Lymph nodes and other sites	36.0 (264) 23.3 (171) 16.6 (122) 24.0 (176)	27.7 (164) 20.5 (121) 26.7 (158) 25.0 (148)	0 > 1 NS 0 < 1 NS
Cycle length			
14-day cycle 21-day cycle 28-day cycle	46.8 (341) 48.1 (351) 5.1 (37)	36.3 (217) 53.6 (320) 10.1 (60)	U, *p* < 0.001
Emetogenicity of the CTX regimen			
Minimal/low Moderate High	17.4 (127) 58.0 (423) 24.6 (179)	22.1 (132) 64.7 (387) 13.2 (79)	U, *p* < 0.001
Antiemetic regimen			
None Steroid alone or serotonin antagonist alone Serotonin antagonist and steroid NK-1 receptor antagonist and two other antiemetics	6.7 (48) 19.3 138) 46.7 (334) 27.3 (195)	7.6 (44) 21.9 (127) 48.9 (284) 21.7 (126)	χ^2^ = 5.71, *p* = 0.126

Abbreviations: CTX = chemotherapy, FE = Fisher’s Exact Test, kg = kilograms, m^2^ = meter squared, MSAS = Memorial Symptom Assessment Scale, NK = neurokinin, NS = not significant, RT = radiation therapy, SD = standard deviation, U = Mann Whitney U test

### Differences in occurrence, severity, and distress ratings

Compared to the younger group (14.8 (± 7.0)), the older group reported a significantly lower mean number of symptoms (12.9 (± 7.2)). Regarding occurrence rates, 18 of the 38 symptoms on the MSAS (47.4%) differed between the two age groups (Table 2). In terms of severity, nine of the 38 symptom scores (23.7%) differed between the age groups. Older patients reported significantly higher severity scores for cough, shortness of breath, and difficulty breathing. In terms of distress, 3 of the 38 symptom scores (7.9%) differed between the age groups. Older patients reported significantly higher distress scores for lack of appetite, cough, and shortness of breath. Table 3 ranks the top ten symptoms based on occurrence, severity, and distress ratings for the two age groups.

**Table 2:** Differences in Symptom Occurrence, Severity, and Distress Between the Two Age Groups

Symptom^a^	Occurrence^b^			Severity^c^					Distress^d^				
	Younger 55.2% *n* = 741	Older 44.8% *n* = 602	*p*-value^e^	Younger		Older		*p*-value^f^	Younger		Older		*p*-value^f^
	%	%		Mean	SD	Mean	SD		Mean	SD	Mean	SD	
Lack of energy	84.5	81.6	0.162	2.02	0.73	2.02	0.72	0.872	1.77	1.16	1.81	1.12	0.613
Difficulty sleeping	72.6	64.8	0.002	2.07	0.80	1.92	0.69	0.010	1.82	1.15	1.73	1.04	0.329
Pain	63.3	56.9	0.021	1.95	0.75	1.88	0.69	0.312	1.84	1.15	1.68	1.02	0.099
Feeling drowsy	60.4	60.1	0.910	1.75	0.70	1.75	0.70	0.878	1.17	1.06	1.15	1.04	0.741
Hair loss	57.5	51.4	0.027	2.50	1.11	2.48	1.15	0.744	1.91	1.36	1.84	1.32	0.531
Difficulty concentrating	57.1	45.6	< 0.001	1.57	0.62	1.52	0.67	0.170	1.48	1.08	1.47	1.06	0.849
Worrying	57.0	46.1	< 0.001	1.87	0.79	1.81	0.66	0.682	1.63	1.12	1.62	0.93	0.729
Nausea	52.7	41.1	< 0.001	1.81	0.81	1.68	0.81	0.025	1.69	1.14	1.58	1.10	0.269
Feeling sad	51.4	39.6	< 0.001	1.76	0.74	1.62	0.63	0.029	1.56	1.10	1.40	0.98	0.136
Numbness/tingling in hands/feet	51.1	53.6	0.378	1.84	0.79	1.85	0.84	0.979	1.52	1.18	1.54	1.18	0.789
Change in the way food tastes	50.7	47.7	0.295	2.13	0.89	2.11	0.89	0.912	1.71	1.27	1.73	1.25	0.816
Feeling irritable	47.5	33.7	< 0.001	1.76	0.74	1.59	0.68	0.011	1.49	1.05	1.42	0.98	0.611
Dry mouth	45.2	45.6	0.912	1.73	0.75	1.74	0.76	0.876	1.18	1.12	1.28	1.12	0.189
Constipation	45.1	41.6	0.202	1.97	0.88	1.98	0.76	0.470	1.72	1.21	1.66	1.12	0.730
Lack of appetite	42.9	39.4	0.218	1.88	0.79	1.98	0.79	0.091	1.19	1.13	1.40	1.08	0.010
Hot flashes	42.2	19.2	< 0.001	1.93	0.78	1.71	0.85	0.002	1.52	1.16	1.17	1.14	0.006
Skin changes	41.9	29.4	< 0.001	1.90	0.83	1.92	0.78	0.641	1.63	1.20	1.66	1.17	0.686
Feeling nervous	41.8	33.4	0.002	1.65	0.71	1.55	0.62	0.188	1.43	1.03	1.38	0.90	0.860
“I don’t look like myself”	40.5	34.6	0.027	2.15	0.95	2.14	0.91	0.974	2.05	1.21	1.88	1.24	0.125
Sweats	39.0	21.7	< 0.001	1.78	0.79	1.77	0.75	0.993	1.32	1.12	1.21	1.01	0.508
Problems with sexual interest or activity	36.8	21.4	< 0.001	2.41	0.97	2.61	0.97	0.058	1.91	1.30	1.79	1.24	0.447
Feeling bloated	36.0	29.5	0.014	1.81	0.75	1.75	0.69	0.483	1.52	1.11	1.59	1.01	0.223
Dizziness	31.4	31.2	1.000	1.55	0.72	1.45	0.65	0.176	1.23	1.10	1.24	0.88	0.498
Cough	30.5	35.1	0.088	1.35	0.57	1.51	0.66	0.010	0.92	1.02	1.13	1.14	0.050
Diarrhea	30.3	28.7	0.546	1.89	0.86	1.83	0.76	0.714	1.41	1.16	1.53	1.08	0.233
Weight gain	27.8	22.4	0.027	1.61	0.76	1.54	0.60	0.887	1.42	1.34	1.29	1.30	0.399
Increased appetite	26.8	24.7	0.379	1.75	0.72	1.76	0.61	0.556	0.94	1.10	0.87	1.13	0.385
Shortness of breath	26.3	27.5	0.619	1.55	0.74	1.81	0.66	< 0.001	1.42	1.05	1.62	1.01	0.016
Itching	25.2	24.4	0.750	1.71	0.75	1.72	0.73	0.822	1.25	1.08	1.33	1.05	0.423
Weight loss	24.7	25.9	0.612	1.54	0.74	1.59	0.68	0.265	0.86	1.12	1.08	1.21	0.091
Abdominal cramps	24.1	20.5	0.129	1.96	0.80	1.73	0.65	0.022	1.63	1.11	1.58	1.05	0.662
Mouth sores	22.5	19.0	0.136	1.67	0.73	1.75	0.77	0.436	1.38	1.07	1.58	1.03	0.071
Difficulty breathing	19.5	20.5	0.630	1.54	0.70	1.76	0.74	0.010	1.57	1.15	1.70	1.09	0.257
Chest tightness	19.0	16.4	0.221	1.50	0.68	1.59	0.65	0.232	1.43	1.05	1.39	0.94	0.950
Difficulty swallowing	15.3	11.9	0.078	1.69	0.79	1.79	0.87	0.549	1.62	1.16	1.69	1.14	0.670
Vomiting	14.5	9.7	0.009	1.84	0.96	1.73	0.76	0.751	1.81	1.20	1.62	1.15	0.327
Swelling of arms or legs	12.9	16.7	0.051	1.88	0.86	1.95	0.81	0.406	1.61	1.16	1.63	1.17	0.888
Problems with urination	11.6	17.0	0.005	1.74	0.78	1.83	0.83	0.484	1.43	1.22	1.59	1.20	0.365

Abbreviation: SD - standard deviation

Symptom severity and distress scores are for those patients who reported the occurrence of the symptom

^a^Symptoms are from the Memorial Symptom Assessment Scale with the addition of the following six symptoms: chest tightness, difficulty breathing, increased appetite, hot flashes, abdominal cramps, and weight gain

^b^Symptoms are listed in descending order of occurrence in reference to the younger group

^c^Severity ratings without zeros: 1 = slight, 2 = moderate, 3 = severe, 4 = very severe

^d^Distress ratings: 0 = not at all, 1 = a little bit, 2 = somewhat, 3 = quite a bit, 4 = very much

^e^Results using Fisher’s Exact test

^f^Results using the Mann Whitney U test

**Table 3:** Rankings of Symptom Occurrence, Severity, and Distress for the Two Age Groups

	Younger Adults				Older Adults		
Symptom Occurrence							
Rank	Symptom	%			Symptom	%	
1	Lack of energy	84.5			Lack of energy	81.6	
2	Difficulty sleeping	72.6			Difficulty sleeping	64.8	
3	Pain	63.3			Feeling drowsy	60.1	
4	Feeling drowsy	60.4			Pain	56.9	
5	Hair loss	57.5			Numbness/tingling in hands/feet	53.6	
6	Difficulty concentrating	57.1			Hair loss	51.4	
7	Worrying	57.0			Change in the way food tastes	47.7	
8	Nausea	52.7			Worrying	46.1	
9	Feeling sad	51.4			Difficulty concentrating	45.6	
9	---------------------------	---			Dry mouth	45.6	
10	Numbness/tingling in hands/feet	51.1			Constipation	41.6	
Symptom Severity^a^							
Rank	Symptom	Mean	SD	Symptom		Mean	SD
1	Hair loss	2.50	1.11	Problems with sexual interest or activity		2.61	0.97
2	Problems with sexual interest or activity	2.41	0.97	Hair loss		2.48	1.15
3	“I don’t look like myself”	2.15	0.95	“I don’t look like myself”		2.14	0.91
4	Change in the way food tastes	2.13	0.89	Change in the way food tastes		2.11	0.89
5	Difficulty sleeping	2.07	0.80	Lack of energy		2.02	0.72
6	Lack of energy	2.02	0.73	Lack of appetite		1.98	0.79
6	---------------------------------	---	---	Constipation		1.98	0.76
7	Constipation	1.97	0.88	Swelling of arms and legs		1.95	0.81
8	---------------------------------	---	---	Skin changes		1.92	0.78
8	Abdominal cramps	1.96	0.80	Difficulty sleeping		1.92	0.69
9	Pain	1.95	0.75	Pain		1.88	0.69
10	Hot flashes	1.93	0.78	Numbness/tingling in hands/feet		1.85	0.84
Symptom Distress^b^							
Rank	Symptom	Mean	SD	Symptom		Mean	SD
1	“I don’t look like myself”	2.05	1.21	“I don’t look like myself”		1.88	1.24
2	Hair loss	1.91	1.36	Hair loss		1.84	1.32
2	Problems with sexual interest or activity	1.91	1.30	---------------------------------		---	---
3	Pain	1.84	1.15	Lack of energy		1.81	1.12
4	Difficulty sleeping	1.82	1.15	Problems with sexual interest or activity		1.79	1.24
5	Vomiting	1.81	1.20	Change in the way food tastes		1.73	1.25
5	---------------------------------	---	---	Difficulty sleeping		1.73	1.04
6	Lack of energy	1.77	1.16	Difficulty breathing		1.70	1.09
7	Constipation	1.72	1.21	Difficulty swallowing		1.69	1.14
8	Change in the way food tastes	1.71	1.27	Pain		1.68	1.02
9	Nausea	1.69	1.14	Constipation		1.66	1.12
9	---------------------------------	---	---	Skin changes		1.66	1.17
10	Worrying	1.63	1.12	Swelling of arms or legs		1.63	1.17
10	Skin changes	1.63	1.20	---------------------------------		---	---

Abbreviation: SD = standard deviation

^a^Severity ratings without zeros: 1 = slight, 2 = moderate, 3 = severe, 4 = very severe

^b^Distress ratings: 0 = not at all, 1 = a little bit, 2 = somewhat, 3 = quite a bit, 4 = very much

### Stability of symptom clusters

Results of the EFAs for younger and older patients are presented in Table 4. For both age groups, an eight-factor solution was selected using the symptom occurrence data. The symptom clusters were named based on the core symptoms within each cluster (i.e., physical and cognitive fatigue, respiratory, psychological, hormonal, chemotherapy-related toxicity, weight gain, gastrointestinal, epithelial). Across the two age groups, the symptom clusters were stable. Subsequent paragraphs describe the consistency of the symptom clusters.

**Table 4:** Comparison of Symptom Clusters Between the Younger and Older Age Groups^a^

Cluster	Symptoms	Younger	Older
Physical and cognitive fatigue symptom cluster	Lack of energy	**0.880**	**0.743**
	Feeling drowsy	**0.621**	**0.463**
	Nausea	**0.430**	−
	Difficulty concentrating	0.401	**0.541**
	Total number of symptoms in this cluster	4	3
	Consistency^b^	2/3	
Respiratory symptom cluster	Difficulty breathing	**1.049**	**0.965**
	Shortness of breath	**0.824**	**0.878**
	Chest tightness	**0.611**	**0.676**
	Cough	−	0.403
	Problems with urination	−	0.402
	Total number of symptoms in this cluster	3	5
	Consistency^b^	3/3	
Psychological symptom cluster	Worrying	**0.792**	**0.812**
	Feeling sad	**0.755**	**0.866**
	Feeling nervous	**0.623**	**0.678**
	“I don’t look like myself”	0.520	−
	Feeling irritable	0.512	0.628
	Difficulty concentrating	0.436	0.437
	Total number of symptoms in this cluster	6	5
	Consistency^b^	3/3	
Hormonal symptom cluster	Hot flashes	**0.807**	**0.728**
	Sweats	**0.788**	**0.783**
	Total number of symptoms in this cluster	2/2	2/2
	Consistency^b^	2/2	
Chemotherapy-related toxicity symptom cluster	Problems with urination	**0.621**	0.418
	Abdominal cramps	**0.481**	**0.489**
	Numbness/tingling in hands/feet	0.466	−
	Mouth sores	−	**0.476**
	Total number of symptoms in this cluster	3	3
	Consistency^b^	1/2	
Weight gain symptom cluster	Weight gain	**0.942**	**0.902**
	Increased appetite	**0.840**	**0.802**
	Total number of symptoms in this cluster	2	2
	Consistency^b^	2/2	
Gastrointestinal symptom cluster	Nausea	−	**0.704**
	Weight loss	**0.548**	−
	Lack of appetite	**0.537**	0.478
	Vomiting	0.526	**0.926**
	Total number of symptoms in this cluster	3	3
	Consistency^b^	0/2	
Epithelial symptom cluster	Change in the way food tastes	**0.709**	**0.612**
	Skin changes	**0.494**	**0.679**
	Hair loss	**0.468**	**0.540**
	“I don’t look like myself”	−	0.522
	Difficulty swallowing	0.464	0.494
	Mouth sores	0.437	−
	Dizziness	−	0.479
	Total number of symptoms in this cluster	5	6
	Consistency^b^	3/3	

^a^Extraction method: unweighted least squares. Rotation method: Geomin (oblique) rotation

^b^Consistency was measured by evaluating whether the same two symptoms (for symptom clusters with a total number of two or three symptoms) or three symptoms (for symptom clusters with a total number of four or more symptoms using the cluster with the highest number of symptoms) with the highest factor loading were present across age groups

− = Factor loadings for these symptoms were < 0.40

Bold font indicates the highest factor loading

### Consistency of the symptom clusters (see table 4)

#### Physical and cognitive fatigue cluster

Physical and cognitive fatigue cluster comprised three (older group) to four (younger group) symptoms. For both age groups, lack of energy had the highest factor loading. Three symptoms (i.e., lack of energy, feeling drowsy, difficulty concentrating) were present in both age groups. However, because the same symptoms did not have the highest factor loadings across the two age groups, this cluster was not consistent.

#### Respiratory cluster

Respiratory cluster comprised three (younger group) to five (older group) symptoms. For both groups, difficulty breathing had the highest factor loading. Three symptoms (i.e., difficulty breathing, shortness of breath, chest tightness) were present in both age groups. Given that the same three symptoms had the highest factor loadings across the two groups, this cluster was consistent.

#### Psychological cluster

Psychological cluster comprised five (older group) to six (younger group) symptoms. Worrying and feeling sad had the highest factor loading for the younger and older groups, respectively. Five symptoms (i.e., worrying, feeling sad, feeling nervous, feeling irritable, difficulty concentrating) were present in both age groups. Given that the same three symptoms had the highest factor loadings across the two groups, this cluster was consistent.

#### Hormonal cluster

Across the two groups, the hormonal cluster consisted of the same two symptoms. Hot flashes and sweats had the highest factor loadings in the younger and older groups, respectively. Given that only two symptoms with the highest factor loadings needed to be present in both groups and hot flashes and sweats had the highest factor loadings across both age groups, this cluster was consistent.

#### Chemotherapy-related toxicity cluster

Chemotherapy-related toxicity cluster consisted of three symptoms. Problems with urination and abdominal cramps had the highest factor loadings in the younger and older age groups, respectively. Two symptoms (i.e., problems with urination, abdominal cramps) were present in both the younger and older groups. Numbness or tingling in the hands or feet was a unique symptom that loaded in the younger group. Mouth sores was a unique symptom that loaded in the older group. Given that the two age groups do not share two symptoms with the highest factor loadings, this cluster was not consistent.

#### Weight gain cluster

Weight gain cluster comprised of the same two symptoms (weight gain, increased appetite) in both age groups. This cluster was consistent.

#### Gastrointestinal cluster

Gastrointestinal cluster comprised four symptoms. Weight loss and vomiting had the highest factor loadings for the younger and older groups, respectively. Given that the two age groups did not share two symptoms with the highest factor loadings, this cluster was not consistent.

#### Epithelial cluster

Epithelial cluster comprised five (younger group) to six (older group) symptoms. Change in the way food tastes and skin changes had the highest factor loadings for the younger and older groups, respectively. Given that three symptoms with highest factor loadings were shared by the two groups, this cluster was consistent.

## Discussion

This study is the first to perform a comprehensive evaluation of multiple dimensions of the symptom experience in younger versus older oncology patients with heterogenous types of cancer and compare the stability and consistency of symptom clusters identified in the two age groups. While both groups experienced a moderate symptom burden [24], compared to the younger group (14.8 (± 7.0)), older adults reported a slightly lower mean number of symptoms (12.9 (± 7.2)). While in previous studies that used the MSAS [25, 26], older adults reported ten concurrent symptoms, the slightly higher number of symptoms in the current study may be related to the larger number of symptoms included on the MSAS (i.e., 32 versus 38); variations in cancer treatments; differences in functional status and/or differences in the number of comorbidities. For example, older patients in current study had a higher KPS score than in previous reports [25, 26], indicating that patients in the current study have a better functional status. This hypothesis is supported by a study of older oncology patients that found that patients with better functional performance had a lower symptom burden [3].

Consistent with previous studies [7, 10], older patients reported significantly lower occurrence rates for almost 50% of the 38 MSAS symptoms. Due to concerns that older oncology patients are at increased risk for chemotherapy-related toxicities [27], they may receive less intense treatment and associated decreases in symptom burden. This hypothesis is supported by the fact that in the current study, the older patients’ MAX2 score was significantly lower which suggests receipt of less toxic chemotherapy regimens, as well as a longer duration between chemotherapy cycles. Another plausible explanation may be that older patients experience an age-related “response shift” that changes their perception of symptoms [28]. Problems with urination was the only symptom that older adults reported a significantly higher occurrence rate. This finding is not surprising given that urinary incontinence is commonly experienced by older adults and is a well-known geriatric syndrome [29].

Consistent with previous studies [7, 10], the most common symptoms in both groups were lack of energy, difficulty sleeping, pain, and feeling drowsy. This finding is not surprising given that these four symptoms are highly prevalent among oncology patients regardless of cancer diagnosis, stage of disease, or treatment modality [30]. In addition, evidence suggests that these symptoms commonly co-occur as part of a “sickness behavior” symptom cluster (i.e., fatigue, pain, disturbed sleep, drowsiness, and lack of appetite) [31].

### Age differences in symptom occurrence, severity, and distress

Eight of thirteen symptoms with the highest occurrence rates were common between younger and older patients (Table 3). While nausea and feeling sad were in the top ten symptoms for younger patients, dry mouth, changes in the way food tastes, and constipation were unique to the older patients. In this study, older patients were more likely to have heart disease, high blood pressure, and lung disease. The fact that many of the medications prescribed for these conditions cause dry mouth may explain the 45.6% occurrence rate for this symptom in our older group [32]. Age-related physiologic changes may explain the high occurrence rates for change in the way food tastes and constipation. For instance, aging is accompanied by gradual loss of taste, that is worsened by polypharmacy and chronic disease [33]. Constipation commonly occurs in older adults due to age-related alterations in the gastrointestinal tract and is worsened by lack of exercise, dehydration, and the occurrence of other chronic conditions [34]. In addition, older adults in the current study were less likely to exercise regularly, which may contribute to the high occurrence rates for constipation.

Consistent with previous studies [7, 26], the symptoms with the highest occurrence rates were not always the most severe or distressing. However, in these two studies [7, 26], for all of the symptoms assessed, older adults reported lower severity and distress ratings. In contrast, in the current study, older patients reported higher severity scores for cough, shortness of breath, and difficulty breathing and higher distress scores for lack of appetite, cough, and shortness of breath. Consistent with a previous report [35], one plausible explanation is that compared to the younger group, older patients in the current study had a significantly higher comorbidity burden and higher rates of lung disease, heart disease, anemia, and lung cancer.

In terms of severity, lack of appetite, swelling of arms and legs, skin changes, and numbness/tingling in hands/feet were in the top ten unique symptoms reported by older adults. Lack of appetite may be related to the high occurrence rates for constipation, change in the way food tastes, and dry mouth in this age group because these symptoms can directly affect appetite and dietary intake [33, 34]. Given that the older patients in this study reported higher rates of heart disease and high blood pressure, swelling of arms and legs may be related to poorer cardiovascular function. In terms of numbness/tingling in hands/feet, older patients may be more susceptible to the neurotoxic effects of chemotherapy and/or pre-existing comorbidities such as diabetes and peripheral vascular disease [36]. This hypothesis is supported by the higher rates of diabetes in our older age group.

In terms of distress, difficulty breathing and difficulty swallowing were in the top ten unique symptoms for the older adults. Difficulty breathing may be explained by the higher rates of lung disease, heart disease, anemia, and lung cancer in the older group. Difficulty swallowing, associated with age-related neuromuscular changes, is a common symptom in older adults [37]. While older adults often successfully adapt to progressive changes in swallowing [37], it is plausible that inflammatory processes associated with chemotherapy may influence this decline in nerve and muscle function. Aging is associated with thinning of the epidermis and dermis, increased water loss, and fragmentation of collagen and elastin [38]. These dermatologic alterations may become more pronounced and distressing during chemotherapy [39].

### Differences in symptom clusters

#### Physical and cognitive fatigue cluster

As noted in a previous report that named the physical and cognitive fatigue cluster, malaise [11], this cluster was not consistent between the two age groups. The fact that nausea loaded only for the younger group may be related to the fact that the younger patients received more toxic and highly emetogenic chemotherapy. While the other three symptoms in this cluster (i.e., lack of energy, feeling drowsy, and difficulty concentrating) were found in both groups their factor loadings differed. Given that lack of energy can be defined as an individual’s potential to perform physical and mental activities [8], the fact that these three symptoms were found in this cluster makes sense clinically.

#### Respiratory cluster

The respiratory cluster was stable and consistent across the two age groups. In a previous study [10], using the original 32 MSAS symptoms, an aerodigestive symptom cluster was identified in only the older patients. While the aerodigestive cluster included cough and shortness of breath, it did not include chest tightness, difficulty breathing, or problems with urination. The fact that chest tightness and difficulty breathing were not included in the original MSAS may explain the differences between the studies.

The occurrence of cough and problems with urination were not found in any of our previous studies of patients with gynecological [40], lung [41], or heterogeneous types of cancer [12] that identified a respiratory symptom cluster. However, in older adults, cough and problems with urination may be explained by the fact that coughing can place pressure on the bladder or prostate gland and make urinary symptoms more pronounced. Given that problems with urination was the only symptom that had a significantly higher occurrence rate in older adults, clinicians should assess for an association with cough, as adequate management of cough may alleviate problems with urination. In addition, in our study of patients with lung cancer [41], cough was a stable and consistent symptom in the respiratory cluster across time and symptom dimensions and was identified in an aerodigestive cluster in older patients [10]. Therefore, additional research is warranted to determine the exact etiologies of cough in older patients with various types of cancer and other comorbid conditions.

#### Psychological cluster

Consistent with previous reports [10, 11], a psychological cluster was identified in both age groups. In one study that used the MSAS [10], compared to nine symptoms for younger patients, fourteen symptoms loaded on this cluster for the older patients. The authors hypothesized that older patients with mood-related complaints may present with various symptoms (i.e., somatic complaints, general aches and pains, hopelessness, anhedonia, depressed mood) that could explain the more diverse nature of this cluster. In another study that used the ESAS to evaluate the severity of nine physical and psychological symptoms [11], a cluster of depression and anxiety was common across the younger and older groups.

A psychological cluster is the most common symptom cluster across a variety of studies of oncology patients [42–45], as well as in our previous studies of patients with breast [46], gastrointestinal [47], gynecological [48], lung [41], and heterogeneous types of cancer [12]. Consistent with our findings and one study of older adults [10], worrying, feeling sad, feeling nervous, and feeling irritable were symptoms with the highest factor loadings across these studies. Given these consistent findings and the high prevalence of depression [49] and anxiety [50] as individual symptoms in oncology patients, clinicians need to routinely assess for these sentinel symptoms and initiate interventions and/or referrals to psychological support services for all oncology patients regardless of age and cancer type.

#### Hormonal cluster

While previous reports of symptom clusters in younger versus older patients did not identify a hormonal cluster in older patients [10, 11], this cluster was stable and consistent across our two age groups. These inconsistent findings may be related to the fact that the original MSAS did not include hot flashes. However, in a study of older breast cancer survivors that evaluated 37 symptoms using the Symptom Bother Scale [5], a hormonal cluster was identified. While hot flashes loaded on this cluster, so did a number of other symptoms not included on the MSAS (e.g., mood swings, nightmares, headaches, vaginal dryness). Given that a variety of cancer treatments, as well as aging, results in changes in sex steroid hormones, additional hormone-related symptoms may warrant evaluation to better elucidate this symptom cluster.

#### Chemotherapy-related toxicity cluster

Chemotherapy-related toxicity cluster was another inconsistent cluster. Of note, numbness/tingling in hands/feet loaded with only the younger group and mouth sores loaded with only the older group. While in a previous study that used the 32-item MSAS, this cluster was stable across the age groups [10], the symptoms in this cluster were different and some were included in other symptom clusters. For example, problems with urination and abdominal cramps, loaded on a genitourinary cluster in older adults and mouth sores loaded with the younger group but not the older group. These differences may be related to physiologic changes associated with aging and/or age-related variations in treatments [27].

#### Weight gain cluster

Given that the original MSAS did not evaluate weight gain and increased appetite, it is not surprising that previous studies did not identify this cluster [5, 6, 10, 11]. In one of the studies of older adults with breast cancer, that evaluated levels of distress associated with weight gain or loss using the Symptom Bother Scale [5], weight gain did not load on any of the clusters identified. In our previous studies, while this cluster, named nutrition or weight change, was stable across time and symptom dimensions in patients with gastrointestinal [51], gynecological [40], lung [41], and heterogeneous types of cancers [12], it was not consistent. In addition, in patients with breast cancer [52], this cluster was neither stable nor consistent. Given the variability of this cluster across cancer types and the paucity of evidence for this cluster in older oncology patients, additional research is warranted to characterize this cluster across different types of cancer in both younger and older oncology patients.

#### Gastrointestinal cluster

Consistent with a previous report [10], the gastrointestinal cluster was not consistent between the two age groups. In addition, consistent with our previous studies [12, 40, 46, 51], the stability and consistency of this cluster was variable across time, symptom dimensions, and cancer types. Differences in comorbid conditions, types of cancer and stages of disease, and previous and current treatments may explain the dynamic nature of this symptom cluster.

#### Epithelial cluster

While the epithelial cluster was stable and consistent across the two age groups, it is interesting to note that “I don’t look like myself” loaded only for the older group. In the younger group, this symptom loaded on the psychological cluster. This finding suggests that this symptom may have different etiologies in older and younger patients and require different interventions. While none of the previous reports in older patients identified this cluster [5, 6, 10, 11], some individual symptoms were included in other symptom clusters. For example, in a study that used the 32 item MSAS [10], while change in the way food tastes and “I don’t look like myself” were included in a nutritional cluster; hair loss, skin changes, and “I don’t look like myself” were included in a chemotherapy toxicity cluster.

In our previous studies of patients with breast [46], gastrointestinal [51], gynecological [40], and lung [41] cancer, this cluster was variable across time and symptom dimensions. Despite this variability, change in the way food tastes and changes in skin were included regardless of cancer type. Given that these two symptoms had the highest factor loadings in both younger and older groups suggests that they may be core or sentinel symptoms in the cluster. Of note, taste changes may contribute to poorer nutritional status that is associated with increased mortality, greater functional decline, and diminished quality of life in older oncology patients [53]. Therefore, clinicians need to assess for these core symptoms and initiate interventions.

### Limitations

Several limitations warrant consideration. Given the study’s cross-sectional design, an examination of age differences in the occurrence, severity, and distress of symptoms, as well as the stability and consistency of symptom clusters across other symptom dimensions (i.e., severity, distress) and time warrant evaluation. Because the sample was primarily well-educated, female, and homogeneous in terms of self-reported race and ethnicity, findings may not generalize across other samples. Because the primary reason for refusal to participate was “being overwhelmed with treatment,” these findings may under-estimate patients’ symptom burden. However, this large sample of oncology patients undergoing chemotherapy, the evaluation of 38 symptoms, the use of EFA to identify the clusters, and the application of rigorous criteria to compare the stability and consistency of the clusters between younger and older age groups are major strengths of this study.

### Conclusions and implications for research and practice

Our findings suggest that eight symptom clusters are stable across younger and older patients. However, three of these clusters (i.e., physical and cognitive fatigue, chemotherapy-related toxicity, gastrointestinal) were not consistent. These inconsistencies may be attributed to aging processes and/or variations in treatments between younger and older oncology patients. Additional research is needed to evaluate the underlying mechanisms associated with these inconsistencies. An increased understanding of these mechanisms will provide direction for symptom management interventions. Given the paucity of research on differences in symptom clusters in younger versus older patients, additional studies are needed to confirm our findings and evaluate for age-related differences in symptom clusters within specific cancer types and/or chemotherapy regimens. In addition, age-related differences in the stability and consistency of symptom clusters across multiple cycles of chemotherapy warrant investigation. Equally important, given the emergence of the use of network analysis to expand our knowledge of the relationships between and among symptom clusters [54] and the use of machine learning methods to predict patient outcomes [55–58], future studies should use these methods to identify patients with a higher symptom burden and initiate timely interventions to prevent or treat symptom clusters.

Findings from this study confirm that older oncology patients experience a moderate to high symptom burden. An examination of age-related differences in common symptoms associated with cancer and its treatments and how these co-occur to form symptom clusters, provide important insights into the symptom experience of younger and older oncology patients. Our findings may help inform future iterations of a comprehensive geriatric assessment that incorporates core and sentinel symptoms unique to older adults with cancer undergoing chemotherapy [59].

## Data Availability

Data are available from the corresponding author following the completion of a data sharing agreement with the University of California, San Francisco.
